# Uncovering the Subtype-Specific Molecular Characteristics of Breast Cancer by Multiomics Analysis of Prognosis-Associated Genes, Driver Genes, Signaling Pathways, and Immune Activity

**DOI:** 10.3389/fcell.2021.689028

**Published:** 2021-07-01

**Authors:** Xinhui Li, Jian Zhou, Mingming Xiao, Lingyu Zhao, Yan Zhao, Shuoshuo Wang, Shuangshu Gao, Yuan Zhuang, Yi Niu, Shijun Li, Xiaobo Li, Yuanyuan Zhu, Minghui Zhang, Jing Tang

**Affiliations:** ^1^College of Bioinformatics Science and Technology, Harbin Medical University, Harbin, China; ^2^School of Medicine, Southern University of Science and Technology, Shenzhen, China; ^3^Department of Pathology, The People’s Hospital of Liaoning Province, Shenyang, China; ^4^Department of Pathology, Harbin Medical University, Harbin, China; ^5^Department of Oncology, Chifeng City Hospital, Chifeng, China; ^6^Department of Pathology, Chifeng City Hospital, Chifeng, China

**Keywords:** breast cancer, molecular subtypes, driver gene, immune infiltration, prognosis

## Abstract

Breast cancer is a heterogeneous malignant disease with different prognoses and has been divided into four molecular subtypes. It is believed that molecular events occurring in breast stem/progenitor cells contribute to the carcinogenesis and development of different breast cancer subtypes. However, these subtype-specific molecular characteristics are largely unknown. In this study, we employed 1217 breast cancer samples from The Cancer Genome Atlas (TCGA) database for a multiomics analysis of the molecular characteristics of different breast cancer subtypes based on PAM50 algorithms. We detected the expression changes of subtype-specific genes and revealed that the expression of particular subtype-specific genes significantly affected prognosis. We also investigated the mutations and copy number variations (CNVs) of breast cancer driver genes and the representative genes of ten signaling pathways in different subtypes and revealed several subtype-specifically altered genes. Moreover, we detected the infiltration of various immune cells in different subtypes of breast cancer and showed that the infiltration levels of major immune cell types are different among these subtypes. Additionally, we investigated the factors affecting the immune infiltration level and the immune cytolytic activity in different breast cancer subtypes, namely, the mutation burden, genome instability and cancer-associated fibroblast (CAF) infiltration. This study may shed light on the molecular events contributing to carcinogenesis and development and provide potential markers and targets for the clinical diagnosis and treatment of different breast cancer subtypes.

## Introduction

Breast cancer is a heterogeneous disease. In 2000, Perou first reported the molecular characteristics-based classification of breast cancer, namely, the luminal subtype (lumA and lumB), basal-like subtype, HER2-overexpression subtype and normal breast-like subtype (Perou et al., 2000). Sorlie et al. (2003) divided the luminal subtype into A type and B/C type. Each subtype has unique molecular signatures, prognoses, clinical behaviors and treatment responses. For example, the prognosis of patients with the lumA and lumB subtypes is relatively good, and the primary treatments are surgery, chemotherapy and endocrine therapy (Harbeck et al., 2019). The 5-year survival rate of patients with the basal-like subtype is low, and there is a lack of effective treatments. Patients with the HER2 subtype are usually treated with targeted drugs or chemoradiotherapy until the tumor has been reduced to a specific size range before undergoing surgical resection (Goldhirsch et al., 2013). Studies have shown that the clinically identified HER2 and basal-like subtypes are complex, explaining why the clinical effects of drugs such as Herceptin on patients with the HER2 subtype are general, and why triple-negative breast cancer is largely difficult to treat (Kumar and Aggarwal, 2016).

At present, numerous studies on breast cancer have focused on tumorigenesis, development, treatment, and improving prognosis (Nagini, 2017; Harbeck et al., 2019; Wang et al., 2019). An increasing number of studies have revealed that the traditional immunohistochemical classification of cancer has some limitations in understanding breast cancer heterogeneity. A more accurate and helpful subtype prediction model of breast cancer is developed through computational biology methods at various molecular levels, which makes up for the lack of immunohistochemical typing. Furthermore, through a deeper mechanism of breast cancer research, reliable and effective treatment regimens can be revealed. However, different molecular subtypes have different effects on treatment and prognosis, and the mechanism remains unclear. Wallden et al. (2015) reported that the PAM50 assay has clinical accuracy and technical precision based on several clinical validation studies. In this project, we used the current academic authoritative PAM50 subtype prediction algorithm to classify breast cancer samples in The Cancer Genome Atlas (TCGA) public database and analyzed the differences among the subtypes at the DNA and RNA levels. We analyzed the difference in driver gene mutations, copy number variation (CNV), and gene fusion in the different subtypes and assessed their prognostic impact. To assess the differences in immune cell levels among the subtypes, we also analyzed the infiltration levels of immune cells and cancer-associated fibroblasts (CAFs). We aimed to determine the potential differences among different subtypes through bioinformatics methods and to find suitable therapeutic targets for clinically conquering breast cancers of different subtypes.

## Materials and Methods

### Data Source

The breast cancer data included 113 normal samples and 1104 tumor samples from TCGA database^1^. We only used samples that had available data from the UCSC Xena database^2^ across the following four genomic platforms: RNA expression, gene mutation, CNV, and gene fusion. The representative genes of ten classic pan-cancer signaling pathways were obtained from the PathwayMapper database^3^ (Bahceci et al., 2017). The driver genes studied in this analysis were predicted by at least seven algorithms (accounting for half of the total number of algorithms) in the DriverDBv3 online database^4^ (Liu et al., 2020).

### Subtype and Prognosis Analysis of Breast Cancer

Gene expression profiles were used to classify the molecular subtypes of the breast cancer samples from the TCGA. The PAM50 algorithm developed by Parker et al. was applied for this process (Parker et al., 2009). The prognostic analysis was performed on samples of the five subtypes obtained (lumA, lumB, HER2, basal-like, and normal) using Kaplan–Meier analysis.

### Screening of Subtype-Specific RNA and Driver Genes in Each Subtype

The transcriptome data of the breast cancer samples from the TCGA were used to perform subtype-specific RNA analyses among the five molecular subtypes. The method used the Seurat3.0 package, and a *p* value less than 0.01 was considered to represent a statistically significant difference between a specific subtype and other subtypes.

We then used the 15 recognized driver gene prediction algorithms provided by the DriverDBv3 database to identify the driver genes. We defined driver genes as genes that were determined to be driver genes by more than seven algorithms.

### DNA-Level Differences Between Subtypes

The DNA-level changes in the driver genes and ten oncogenic pathway representative genes were analyzed in our study, namely, CNV, gene mutation, and gene fusion. The frequency of DNA changes and the number of samples with DNA changes were analyzed in detail for each subtype.

### Analysis of Tumor Mutation Burden

Tumor mutation burden (TMB) was defined as the total amount of somatic gene coding errors, base substitutions, insertions or deletions detected per million bases. TMB data were downloaded from the TCGA database through the GDC tool. We classified the samples of each subtype into low-and high-TMB groups according to the median data. Then, we merged the TMB data with corresponding survival information via the ID number of the samples. Kaplan–Meier analysis was conducted to compare the survival difference between the low-and high-TMB groups of each subtype, and the *p* value of the log-rank test was calculated.

### Selection and Classification of Genes in Pathways

Genes were assigned to pathways based on a combined revision of pathway analyses from previous papers published between 2008 and 2017, a review of the scientific literature and expert curation. Several genes in the pathways, such as TGF-β, Myc, and PI3K, had been analyzed by specific working groups. These groups were led by experts on each pathway, and each published separate manuscripts (Foroutan et al., 2017; Ge et al., 2018; Knijnenburg et al., 2018; Peng et al., 2018). The pathways included in our study were (1) Hippo signaling, (2) cell cycle, (3) TGFβ signaling, (4) Notch signaling, (5) receptor tyrosine kinase (RTK)/RAS signaling, (6) β-catenin/Wnt signaling, (7) oxidative stress response/Nrf2, (8) Myc signaling, (9) P53, and (10) PI-3-kinase signaling. Gene mutation, copy number amplification and deletion were evaluated in all essential representative genes from the 10 pathways. The frequency of crucial gene changes in the pathways was also counted.

### Infiltration of Immune Cells and Fibroblasts

The infiltration levels of immune cells and fibroblasts in breast cancer samples were predicted by Newman et al. (2015) and Becht et al. (2016) algorithms, respectively, using the mRNA expression profiles of the samples. The correlation between immune cells and fibroblasts was calculated by Pearson correlation. All the data processing was performed using R 3.6.1.

### Immunophenoscore Analysis in Breast Cancer

An immunophenogram was used to predict anti-PD-1/PD-L1 therapy responses across cancers (Charoentong et al., 2017). The immunophenoscore (IPS) was calculated by the immunophenogram among four cancer subtypes (CTLA4_negative + PD-1_negative, CTLA4_positive + PD-1_negative, CTLA4_ negative + PD-1_ positive, CTLA4_ positive + PD-1_positive) from the TCGA-BRCA database. The IPS ranged from 0 to 10. A high PD-1_positive IPS indicated a well-predicted response to anti-PD-1/PD-L1 therapy. The R code used is available at GitHub^5^.

## Results

### Identification of Molecular Subtype-Specific RNA in Breast Cancer

**FIGURE 1:**
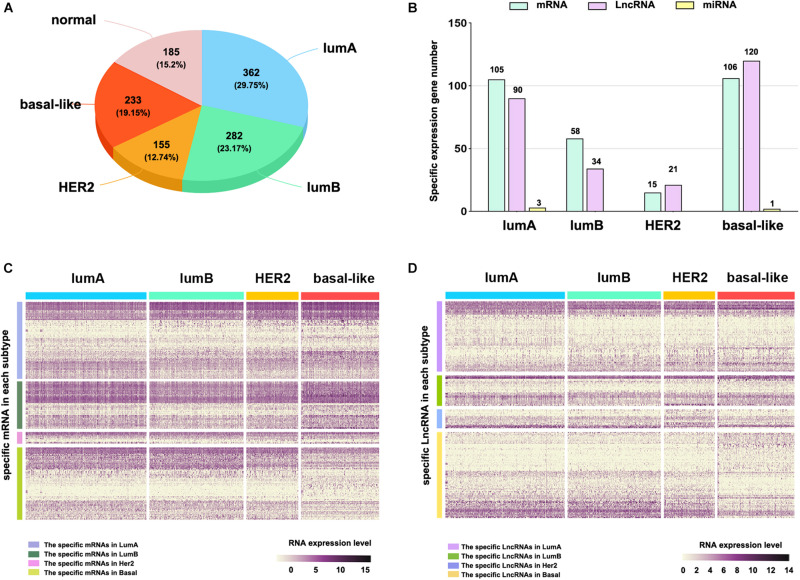
The subtype-specific RNA expression. **(A)** The number of samples of each breast cancer subtype. **(B)** The number of subtype-specific RNAs in each subtype. **(C)** The expression of specific mRNAs in each subtype. **(D)** The expression of specific lncRNAs in each subtype.

**FIGURE 2:**
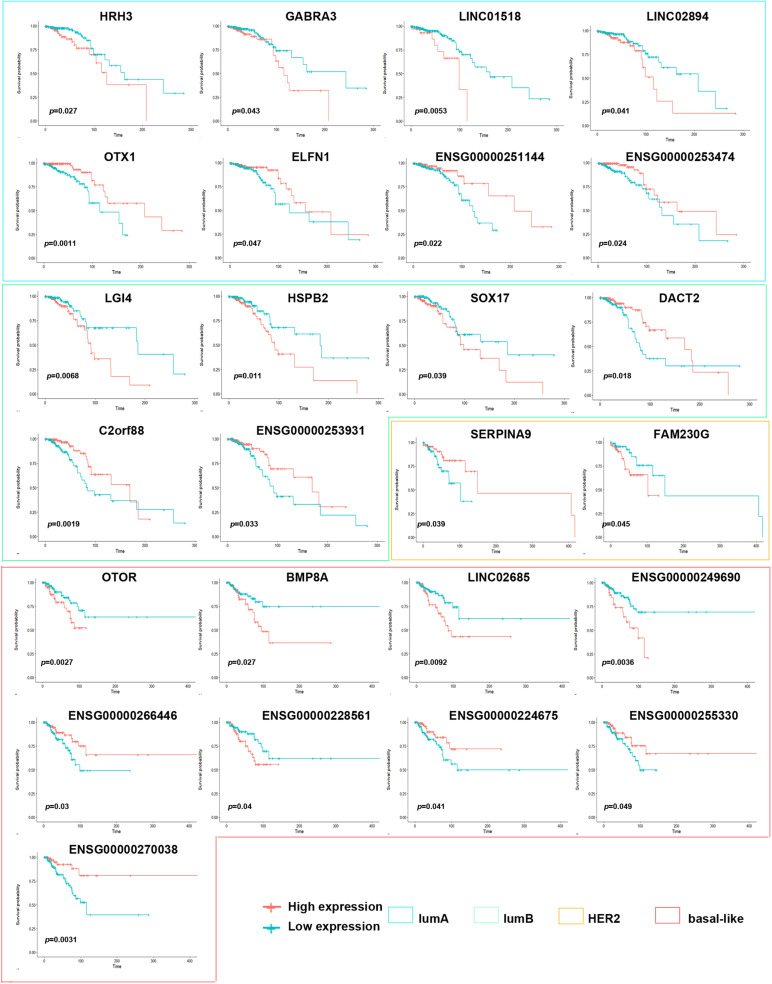
Correlation of expression of subtype-specific RNA with overall survival in breast cancer. Kaplan–Meier survival curves were generated for subtype-specific RNA by comparing groups of high (red line) and low (blue line) gene expression. *p* < 0.05 in log-rank test.

To explore the differences among breast cancer molecular subtypes, we first used the PAM50 method to predict the molecular subtypes of 1,217 breast cancer samples from the TCGA. The prediction results showed that the number of samples in each breast cancer subtype was lumA: 362, lumB: 282, HER2: 155, basal-like: 233, and normal: 185 (Figure 1A). We counted the number of RNAs specifically expressed in each subtype, as shown in Figure 1B (Supplementary Table 1). We compared differential expression levels of mRNA, lncRNA and miRNA in each subtype (Figures 1C,D and Supplementary Figure 1). We generated Kaplan–Meier survival curves to explore the potential link between subtype-specific RNA and OS. The median was used as the cutoff for high or low expression chosen for subtype-specific RNA. Among the RNAs, a total of 25 were shown to significantly predict OS (Figure 2, *p* < 0.05). These genes were considered to be potential subtype-related prognostic genes.

### Gene Mutations Among the Breast Cancer Subtypes

**FIGURE 3:**
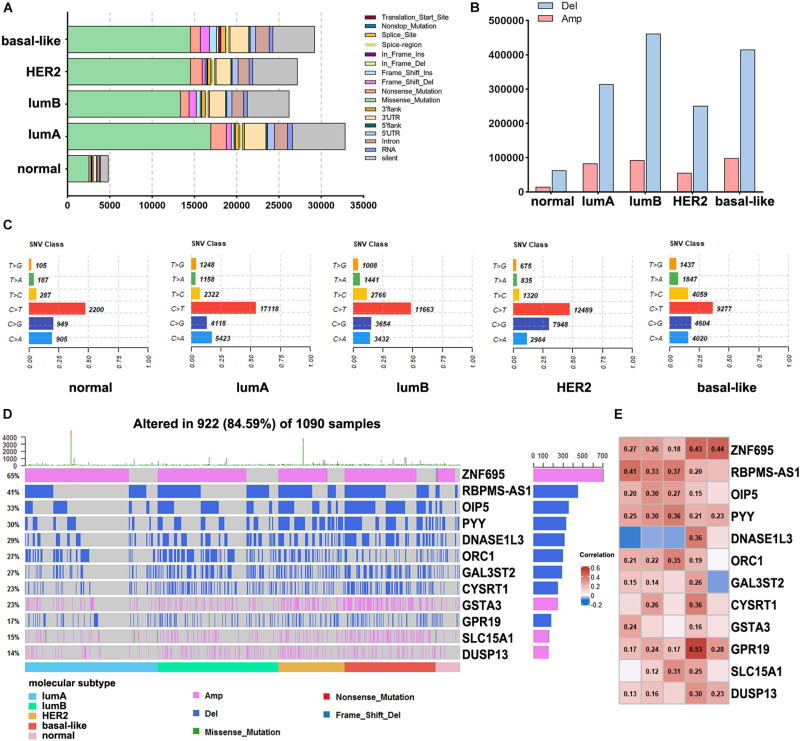
Analysis of DNA alterations in different breast cancer subtypes. **(A)** The different classified categories of DNA mutations in each breast cancer subtype. **(B)** The number of copy number variations in each subtype. **(C)** Statistics of SNV changes in different subtypes. **(D)** Waterfall chart showing the mutations of different subtype-specific genes. **(E)** Correlation analysis of subtype-specific RNAs and their modifications in different subtypes. The numbers in the figure are correlation coefficients, a negative value represents a negative correlation, and a positive value represents a positive correlation.

To assess the alteration of genes among different subtypes in breast cancer, we analyzed the mutations in each subtype, including gene mutation and CNV. In summary, these mutations were classified according to different categories, in which missense mutations accounted for the largest fraction (Figure 3A). The lumA subtype had the most missense mutations, and the lumB subtype had the lowest (16,967 and 13,364 mutations, respectively). The CNV analysis across the five subtypes revealed that the highest levels of amplification and deletion were detected in the basal-like and lumB subtypes (Figure 3B). Comparison across the five subtypes revealed that they all had increased C > T transversions (Figure 3C). The C > G transversions were markedly higher in the HER2 subtype than in other subtypes. The basal-like subtype had more T > C transversions than the other subtypes. We further performed mutation analysis on the subtype-specific genes in which mRNA expression was significantly different in each subtype. The results showed that the mutation of these genes, namely ZNF695, RBPMS1-AS1, OIP5, and PYY, correlated with their RNA expression levels (Figures 3D,E).

### Identification of Driver Genes in Breast Cancer Subtypes

**FIGURE 4:**
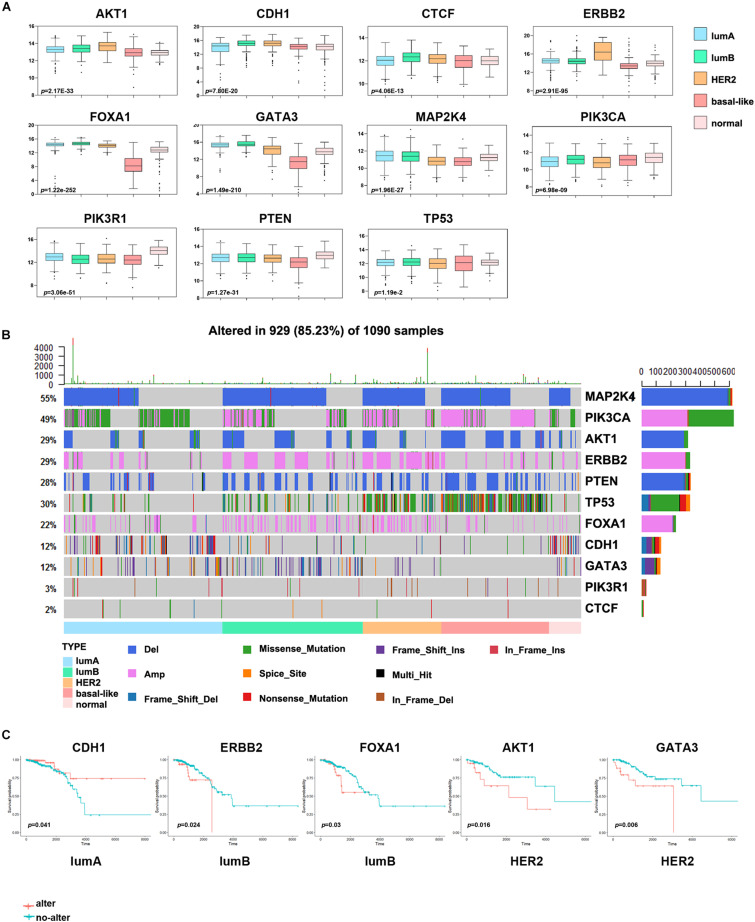
Analysis of the driver genes in different breast cancer subtypes. **(A)** The expression levels of eleven driver genes in each subtype. **(B)** The waterfall chart shows the frequency mutations of the driver genes in each subtype. **(C)** Kaplan–Meier survival curve of the driver genes with gene mutations. Red indicates the gene alteration group, and blue indicates the non-alteration group.

To further understand the molecular features of different subtypes, we used the DriverDBv3 online database to predict the driver genes of breast cancer samples in the TCGA database. To increase the accuracy of our results, we used more than seven algorithms to simultaneously predict driver genes and obtained 11 driver genes, namely ERBB2, AKT1, PIK3CA, PIK3R1, PTEN, TP53, CDH1, GATA3, MAP2K4, CTCF, and FOXA1. We analyzed the effect of driver gene expression in the different subtypes and found that the expression of PIK3CA, PIK3R1, and PTEN was significantly lower in tumor samples than in normal samples (Figure 4A). ERBB2, GATA3, PIK3CA, MAP2K4, and other driver genes are either overexpressed considerably or expressed at low levels in specific subtypes, which indicates that these genes have essential significance in the formation and progression of tumors in these subtypes and may be used as subtype-determining markers and breast cancer treatment targets.

Other studies have also shown that breast cancer cells often have many driver gene mutations (Kaur et al., 2018). To more intuitively show the impact of driver genes on the prognosis of patients with breast cancer, we analyzed the driver gene mutations in each subtype and the abnormal alterations of frequently altered genes and essential cancer genes in different signaling pathways. A large proportion of samples from patients with various subtypes of breast cancer had abnormal changes in the MAP2K4, TP53 and PIK3CA genes (Figure 4B). The change frequency of TP53 in basal-like samples was more significant than that in other subtypes, and missense mutation of PIK3CA was more extensive in the lumA subtype. In addition, many patients with the HER2 subtype had ERBB2 gene alterations, and patients with the lumB subtype had a deletion of MAP2K4. And we discovered that some key oncogenes and tumor suppressor genes in 10 classical signaling pathways, namely MDM4, MTOR, MYC, CCND1, and RB1, had a higher proportion of mutations. Consistent with other studies, the average mutation rate of oncogene PIK3CA and tumor suppressor gene TP53 was as high as 10% (Supplementary Figure 2).

Next, we evaluated the impact of driver gene alterations on the prognosis of patients with breast cancer. We divided the samples of each subtype into an altered group and a non-altered group according to whether the driver gene had a change in mutation, CNV, or gene fusion. This analysis found that the survival effect of the abnormally altered ERBB2 and FOXA1 groups in the lumB subtype group was significantly lower than that of the non-altered group, and the survival effect of the abnormally altered AKT1 and GATA3 groups in the HER2 subtype group was substantially lower than that of the non-altered group (Figure 4C). The survival effect of the abnormally altered CDH1 group in the lumA subtype group was significantly higher than that of the non-altered group.

### Differences in Immune Cell Infiltration of Different Subtypes

**FIGURE 5:**
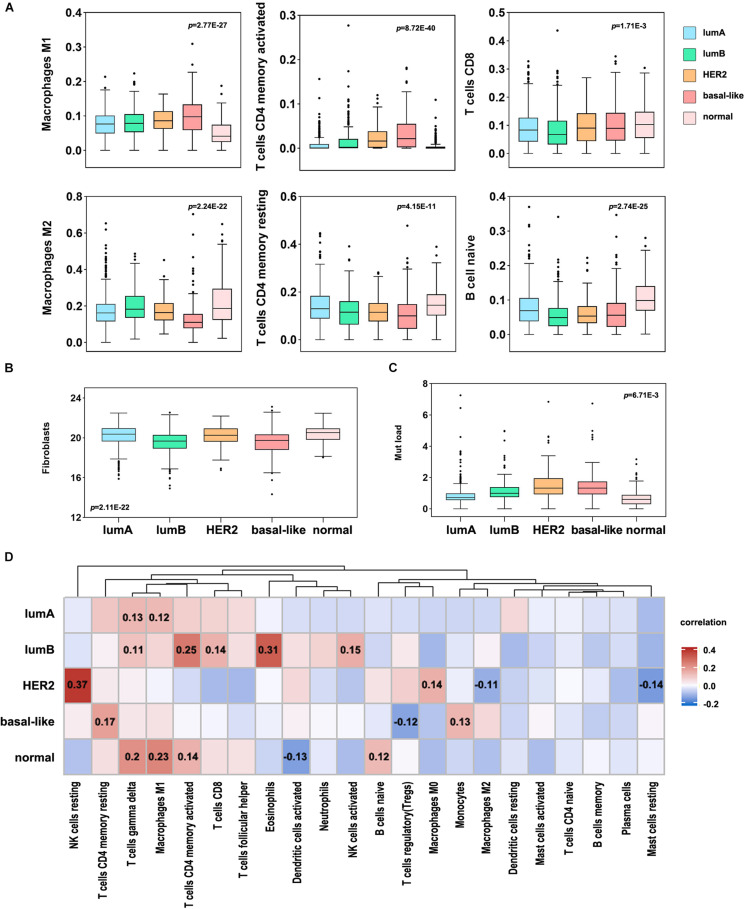
Immune cell infiltration levels in different breast cancer subtypes. **(A)** Differential analysis of the immune cell infiltration level in different breast cancer subtypes (*p* < 0.05). **(B)** The infiltration level of fibroblasts in different breast cancer subtypes. **(C)** Analysis of TMB in different breast cancer subtypes. **(D)** Correlation analysis between TMB and 22 immune cell infiltration levels in different subtypes. Red indicates a positive correlation, and blue indicates a negative correlation. The number represents the degree of correlation, and *p* < 0.05.

A high number of mutations in breast cancer samples indicates inferior genome stability, and many mutations in tumor tissues can induce the production of new antigens. Simultaneously, patients with specific gene mutations are suitable candidates for immunotherapy, such as patients with BRCA1/2 gene mutations (Mateo et al., 2019). Consequently, to study the difference in immune cell infiltration among breast cancer subtypes and identify personalized immunotherapy for patients. Here, we used CIBERSORT to analyze the differences in the infiltration of 22 immune cell types in each subtype of tumor tissues. In the more malignant tumor tissues of the basal-like and HER2 subtypes, the infiltration level of M1 macrophages, activated memory CD4 T cells, and CD8 T cells was significantly higher. In contrast, the infiltration levels of M2 macrophages, naive B cells, and resting memory CD4 T cells were substantially lower (Figure 5A). Then, we used an MCP counter to analyze the fibroblast infiltration levels in each subtype (Figure 5B), among which the infiltration levels of fibroblasts in the lumB and basal-like subtypes were low. Correlation analysis between immune cells and fibroblasts showed that T cells and fibroblast levels were negatively correlated (Supplementary Figure 3).

Furthermore, we analyzed the correlation between TMB and immune cell infiltration in each subtype and found that HER2 mutation was significantly correlated with NK cell infiltration, and the other subtypes were significantly correlated with T cell infiltration (Figures 5C,D). The basal-like subtype was considerably correlated with CD4 T cell infiltration, and the lumB subtype was significantly correlated with CD4 and CD8 infiltration. This suggests that basal-like and lumB subtype tumors with higher TMB have more T cell infiltration and lower fibroblast infiltration. We speculate that the cellular components involved in positive and negative immune responses are complex, leading to poor immunotherapy effects. The proportions of these immune responses could potentially be changed. The components of the positive immune response can work to inhibit negative immune cells, thereby improving the efficacy of immunotherapy for this type of tumor.

**FIGURE 6:**
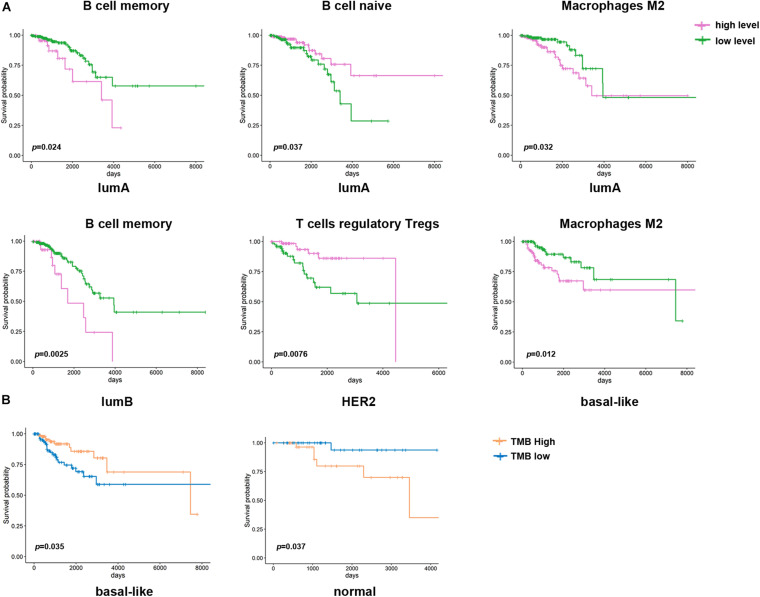
Kaplan–Meier survival curve of immune cells and TMB in different breast cancer subtypes. **(A)** Kaplan–Meier survival curve of the immune cell infiltration level in different breast cancer subtypes (*p* < 0.05). Purple represents a high level of infiltration, and green represents a low level of infiltration. **(B)** Kaplan–Meier survival curve of TMB in different breast cancer subtypes. Blue represents the high TMB group, and orange represents the low TMB group.

Then, we assessed the correlation between immune cell infiltration and prognosis among the different subtypes (Figure 6A). It was shown that a low degree of memory B cell infiltration indicated a better prognosis in the lumA and lumB subtype groups, while naive B cell infiltration indicated a poorer prognosis in the lumA subtype group. The group with high Treg cell infiltration had a more extended survival period in the HER2 subtype group; high M2 macrophage infiltration in the basal-like subtype group had a worse prognosis. The above results suggest that the immune cell infiltration in different subtypes is related to patient prognosis.

### Different Responses to Immunotherapy Among the Different Molecular Subtypes

**FIGURE 7:**
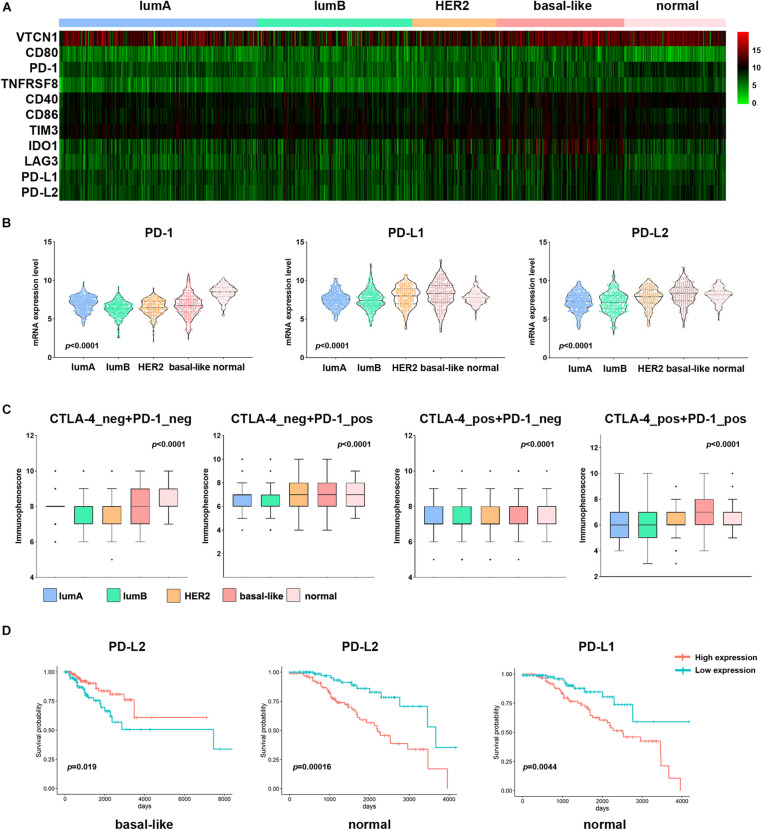
Association of PD-L1 expression and IPS among subtypes in patients with breast cancer. **(A)** Heatmap representation of differences in mRNA expression levels of immune inhibitory checkpoint-related genes. **(B)** Comparison of PD-1, PD-L1, and PD-L2 expression levels between each subtype in breast cancer. **(C)** IPS comparison between each subtype in breast cancer patients in the CTLA4-negative/positive or PD-1-negative/positive groups. CTLA4-positive or PD1-positive represented anti-CTLA4 or anti-PD-1/PD-L1 therapy, respectively. **(D)** Kaplan–Meier survival curves of PD-L1 and PD-L2 in different breast cancer subtypes. Red represents the high expression group, and blue represents the low expression group.

To evaluate the different responses to immunotherapy among the subtypes, we analyzed the mRNA expression of breast cancer immune checkpoint genes, namely PD-1, PD-L1, PD-L2, LAG3, VTCN1, IDO1, and TIM3 (Figure 7A). The expression of PD-L1 and PD-L2 was significantly higher in the basal-like subtype than in other subtypes (Figure 7B). The expression of other immune checkpoint genes (CD40, CD80, CD86, IDO1, and LAG3) was also significantly elevated in the basal-like subtype (Figure 7A and Supplementary Figure 4). VTCN1 and TIM3 expression was higher in the lumA and HER2 subtypes, respectively (*p* < 0.0001). Then, we used immunophenogram analysis to predict the response to anti-PD-1/PD-L1 therapy among the subtypes. We found that in the CTLA4_negative + PD-1_negative subtype, the lumB and HER2 subtypes exhibited a lower IPS than the other subtypes (Figure 7C). In the CTLA4_negative + PD-1_positive and CTLA4_positive + PD-1_positive subtypes, the IPS of the basal-like subtype was significantly higher (Figure 7C). We assessed the correlation between immune checkpoint gene expression and prognosis among the different subtypes. The results showed that patients with the basal-like subtype with higher levels of PD-L2 expression had a better OS rate (Figure 7D). These results indicated that patients with the basal-like subtypes were likely to have a higher positive response to anti-PD-1/PD-L1 therapy or a combination of anti-PD-1/PD-L1 and anti-CTLA4 treatments.

## Discussion

Breast cancer is a heterogeneous disease with different molecular characteristics and various clinical treatment responses and prognoses. This study found that the level of subtype-specific RNA expression can assess patient prognosis within different subtypes. For example, HRH3 and GABRA3 expression was lower in the lumA subtype than in the other subtypes, and the better prognosis of patients with the lumA subtype with low HRH3 and GABRA3 expression. Similarly, Kiranmai Gumireddy et al. reported that high expression of GABRA3 was inversely correlated with breast cancer survival. GABRA3 can promote breast cancer cell migration, invasion and metastasis by activating the AKT pathway (Gumireddy et al., 2016). However, an A-to-I RNA-edited form of GABRA3 showed that edited GABRA3 suppresses breast cancer cell invasion and metastasis. This is the first report in which an edited RNA was found to play a crucial role in the progression, invasion and metastasis of breast cancer, and it may be a potential therapeutic target. Therefore, it is suggested that subtype-specific RNA expression in breast cancer may be targeted therapeutically, along with providing information regarding the prognosis of patients with different breast cancer subtypes.

Driver genes play an essential role in tumor progression. There is apparent heterogeneity in the frequency and diversity of tumor driver gene mutations. Stephens et al. (2012) reported that few driver genes have high-frequency repeated mutations in breast cancer, except PIK3CA and TP53, which is mutated in approximately 30% of tumors, and ERBB2, FGFR1, and CCND1, which are amplified in approximately 15% of tumors. Significantly, even small numbers of driver gene mutations might correlate with the clinical response of breast cancer. In this study, we found that MAP2K4 (55%), PIK3CA (49%), and TP53 (30%) were mutated in a large number of cancer samples. Among them, the level of TP53 mutation was significantly higher in the basal-like subtype than in the other subtypes, which may be the reason for the higher malignancy of TNBC. In addition, the copy number amplification of ERBB2 in the HER2 subtype was significantly higher than that in the other subtypes, which may promote the development of the HER2 subtype. The driver genes abnormally altered in different subtypes may help explain the differences in response to clinical treatment. Our results were consistent with Shah et al. (2012), who reported that in primary TNBCs, TP53 mutation was the most frequent clonal event (53.8%), followed by PIK3CA mutations (10.7%). The mutation frequencies of TP53 are 12–29 and 72–80% in the lumB, HER2, and basal-like subtypes, respectively. This indicates that TP53 mutation plays a critical carcinogenic effect in most breast cancers (Cancer Genome Atlas Network (CGAN)., 2012). Other studies also reported that the frequency of PIK3CA mutations in ER + and HER2 + tumors is 29–45 and 22.7–39%, respectively (Yang et al., 2016). Although the mutation frequency is significantly different, highly active PI3K pathway expression suggests that PI3K inhibitors may be an effective targeted treatment for breast cancer. Some studies have shown that many genes, such as CDK4, MDM2, and CDH1, have significant CNV. Therefore, further research is needed to understand the driver gene mutations in breast cancer, which may have substantial value for targeted therapy (Luen et al., 2016).

In the past, breast cancer was considered a tumor type with poor immunogenicity. Compared with other cancers with a high mutation load that respond well to immunotherapy (such as non-small cell lung cancer and melanoma), breast cancer has a lower mutation load (Smith et al., 2019). However, recent studies have revealed that some molecular subtypes of breast cancer are infiltrated by immune cells, suggesting that immunotherapy may improve the prognosis of these patients (Dieci et al., 2018). Therefore, it is essential to predict whether patients will respond to immunotherapy. In this study, we found that different infiltration levels of immune cells are related to the prognosis of breast cancer. The high infiltration level of M2 macrophages indicated a worse prognosis in the lumA and basal-like subtypes (Figure 6A). The high level of Treg cell infiltration in the HER2 subtype indicates a longer survival time. However, it is generally recognized that Treg cells have a high potential to suppress the immune system. They promote tumor development by inhibiting the effective antitumor immune mechanism of malignant tumors. Therefore, most studies have demonstrated that Treg cell accumulation in breast cancer tumors is related to shorter overall survival times. In addition, we found that the CAF and TMB were also different among the breast cancer subtypes, and both were correlated with immune cell infiltration. In all subtypes of breast cancer, CAFs are negatively correlated with T cell infiltration. In the basal-like subtype, TMB and CD4 + T cell infiltration were higher, CAFs were lower, and high TMB indicated a better prognosis (Figure 6B).

Recent years, PD1 and PDL1 are hot spots in the immune regulation. Several studies reported that they governed pathways acting as feedback to prevent excessive T cell response (Yang et al., 2019). In this study, the basal-like subtype samples showed higher expression of immune checkpoint genes (PD-L1, PD-L2, CD40, CD80, CD86, IDO1, and LAG3) than other subtypes. Immunophenogram analysis of the different subtypes also showed that the basal-like subtype responds well to anti-PD-1 therapy but not to anti-CTLA4 therapy. These features suggest that patients with the basal-like subtype may respond better to immunotherapy. Other studies on TMB and immune cell infiltration also showed that higher TMB tends to promote T cell and NK cell infiltration. Patients with bladder cancer with higher TMB levels have a better prognosis (Fan et al., 2020; Mi et al., 2020; Wu et al., 2020). However, in head and neck squamous cell carcinoma and melanoma, high TMB can lead to a shorter survival period, which may be related to infiltrating immune cells (Jiang et al., 2020, 2021). The above results indicate that the cellular components of the tumor involved in the immune response are complex, which leads to different immunotherapy effects in patients. In the future, it may be possible to change the ratio of immune cells to increase the positive immune cell component and suppress negative immune cells, thereby improving the effect of tumor immunotherapy.

Several recent retrospective and prospective studies have shown that the classification of molecular subtypes and the mechanisms of interaction between tumors and immune cells of different subtypes are significant for predicting therapeutic response and prognosis and developing individualized treatment plans (Waks and Winer, 2019). Therefore, we analyzed the specific molecular characteristics of different subtypes of breast cancer from a multiomics perspective, providing a theoretical basis for selecting patients most likely to benefit from immunotherapy and providing potential biomarkers for future treatments.

## Data Availability Statement

The datasets presented in this study can be found in online repositories. The names of the repository/repositories and accession number(s) can be found in the article/Supplementary Material.

## Author Contributions

XbL, YyZ, MZ, and JT designed and supervised this study. XhL, JZ, and JT drafted the manuscript. SW, LZ, and SG illustrated the figures for the manuscript. YZha, YN, and MX searched the database. XhL, SW, and YZhu analyzed the data. All authors approved the final manuscript.

## Conflict of Interest

The authors declare that the research was conducted in the absence of any commercial or financial relationships that could be construed as a potential conflict of interest.

## Footnotes

1 https://portal.gdc.cancer.gov

2 https://xenabrowser.net/datapages/

3 http://www.pathwaymapper.org/

4 http://ngs.ym.edu.tw/driverdb

5 https://github.com/Mayer/C-imed/Immunophenogram

## Supplementary Material

Supplementary Figure 1 Comparison of specific miRNA expression levels among the breast cancer subtypes.

Supplementary Figure 2 Mutation frequency of key genes in ten classic signaling pathways. The three black dots on the left side of the gene name represent gene mutation, copy number amplification and deletion. The colors represent oncogenes and tumor suppressor genes. The intensity of the color represents the frequency of mutations.

Supplementary Figure 3 Correlation analysis of fibroblast infiltration levels and twenty-two immune cell infiltration levels in different subtypes. Red indicates a positive correlation, and blue indicates a negative correlation. The number represents the degree of correlation, and *p* < 0.05.

Supplementary Figure 4 Comparison of immune checkpoint gene mRNA expression levels among the breast cancer subtypes.
