# Supplementary figures and images for: Uncovering the Subtype-Specific Molecular Characteristics of Breast Cancer by Multiomics Analysis of Prognosis-Associated Genes, Driver Genes, Signaling Pathways, and Immune Activity
Source: Front Cell Dev Biol. 2021 Jul 1;9:689028. doi: 10.3389/fcell.2021.689028 (PMC8280810; doi:10.3389/fcell.2021.689028)

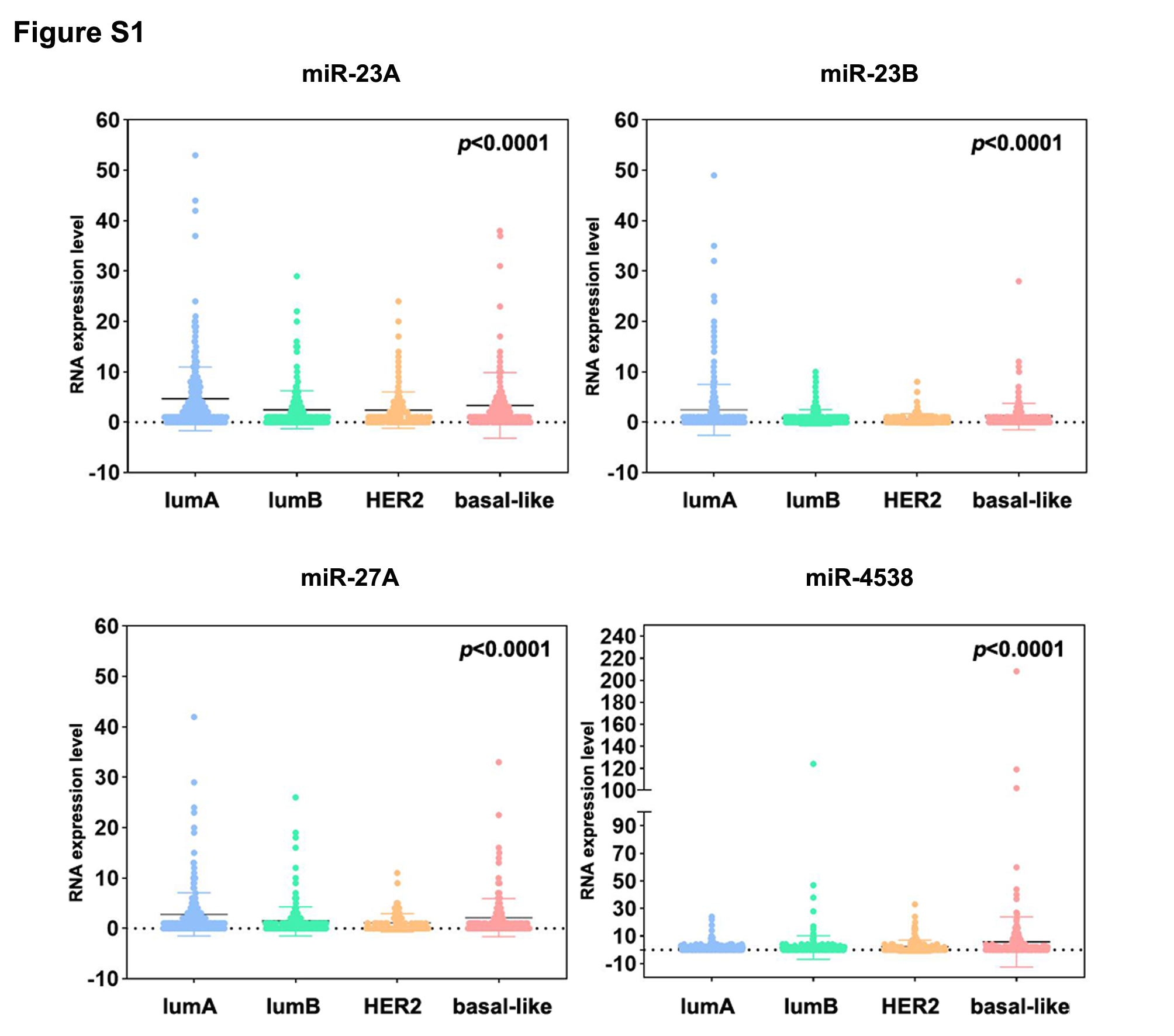

Supplement: Supplementary Figure 1 — Comparison of specific miRNA expression levels among the breast cancer subtypes. [file Image_1.TIF]

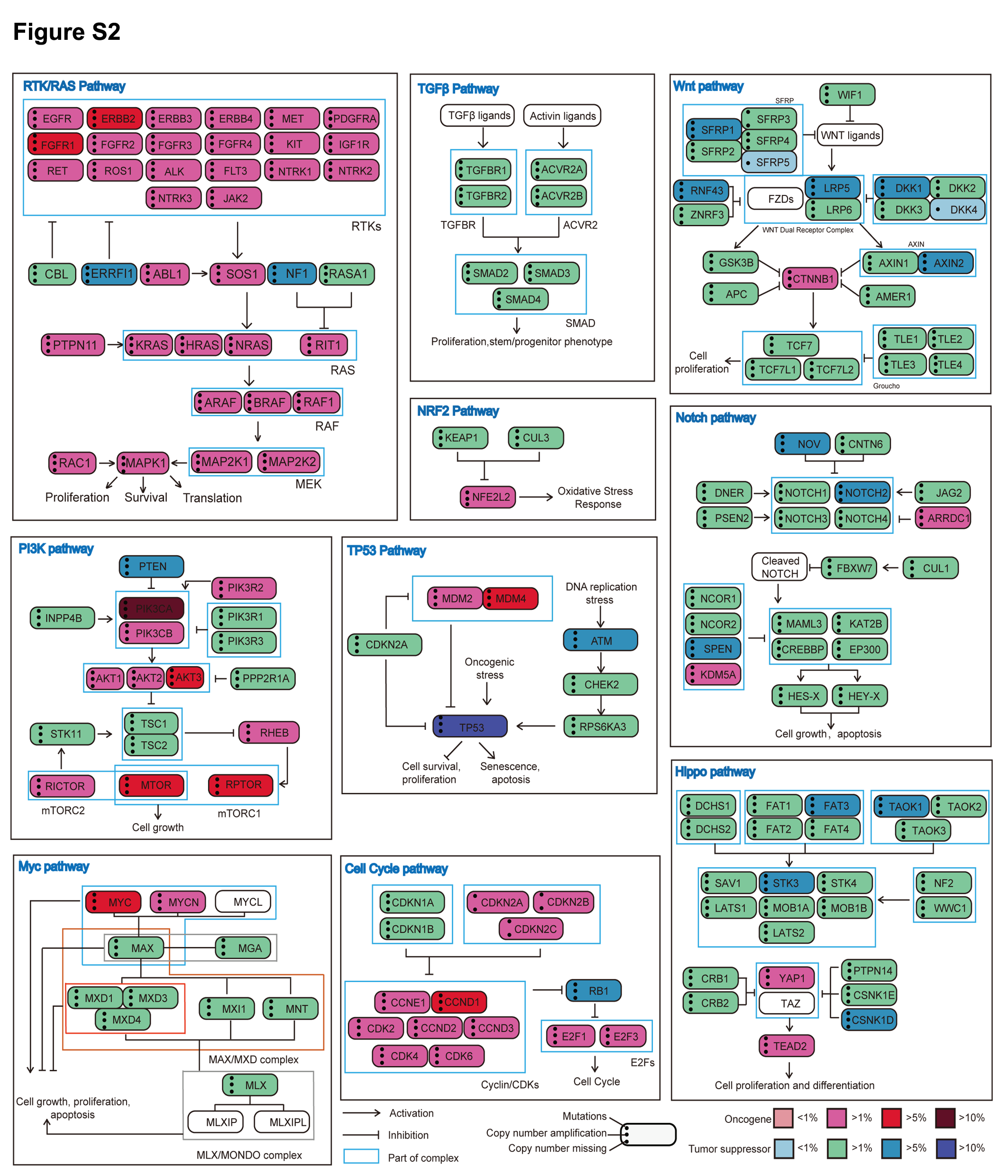

Supplement: Supplementary Figure 2 — Mutation frequency of key genes in ten classic signaling pathways. The three black dots on the left side of the gene name represent gene mutation, copy number amplification and deletion. The colors represent oncogenes and tumor suppressor genes. The intensity of the color represents the frequency of mutations. [file Image_2.TIF]

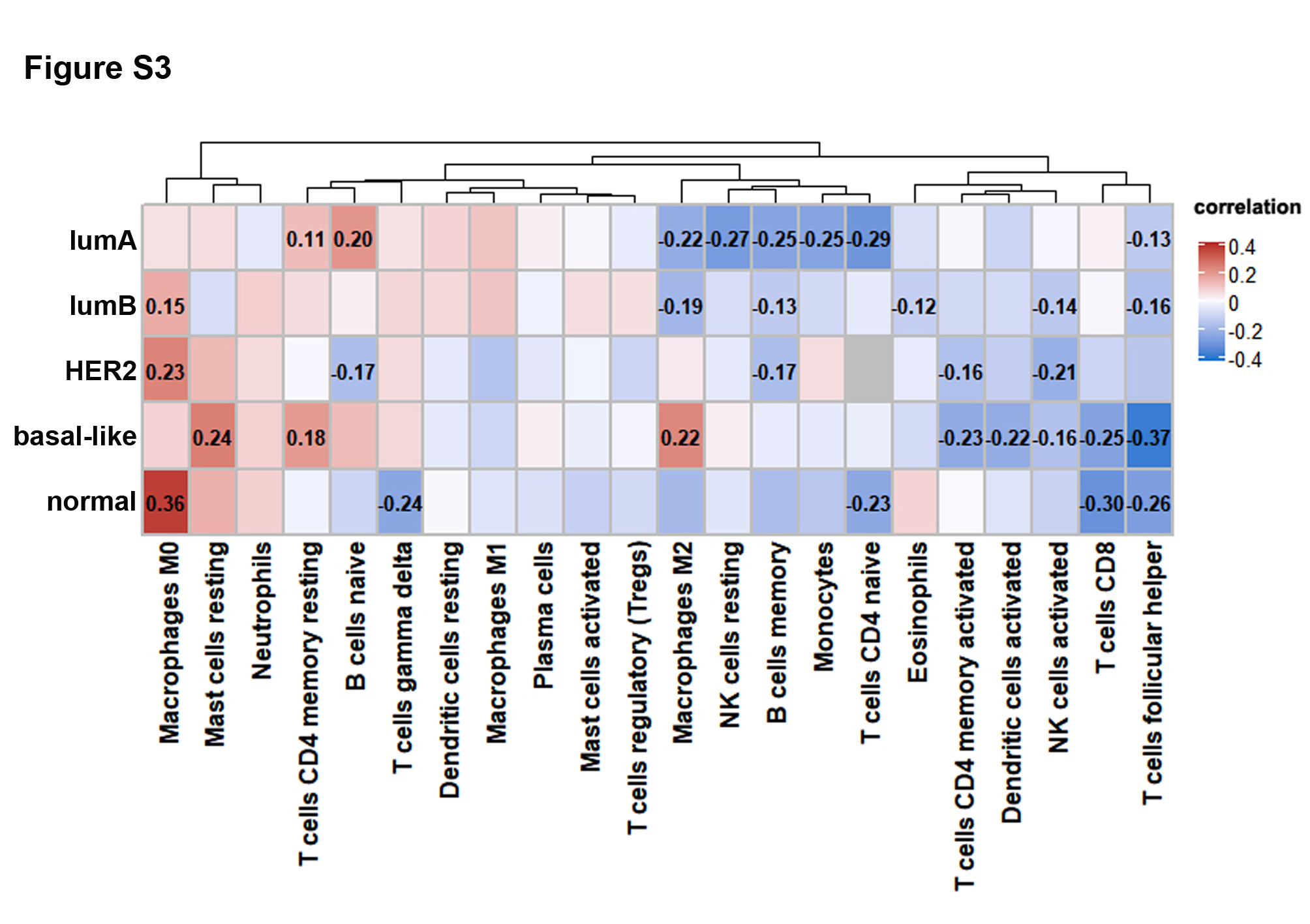

Supplement: Supplementary Figure 3 — Correlation analysis of fibroblast infiltration levels and twenty-two immune cell infiltration levels in different subtypes. Red indicates a positive correlation, and blue indicates a negative correlation. The number represents the degree of correlation, and p < 0.05. [file Image_3.TIF]

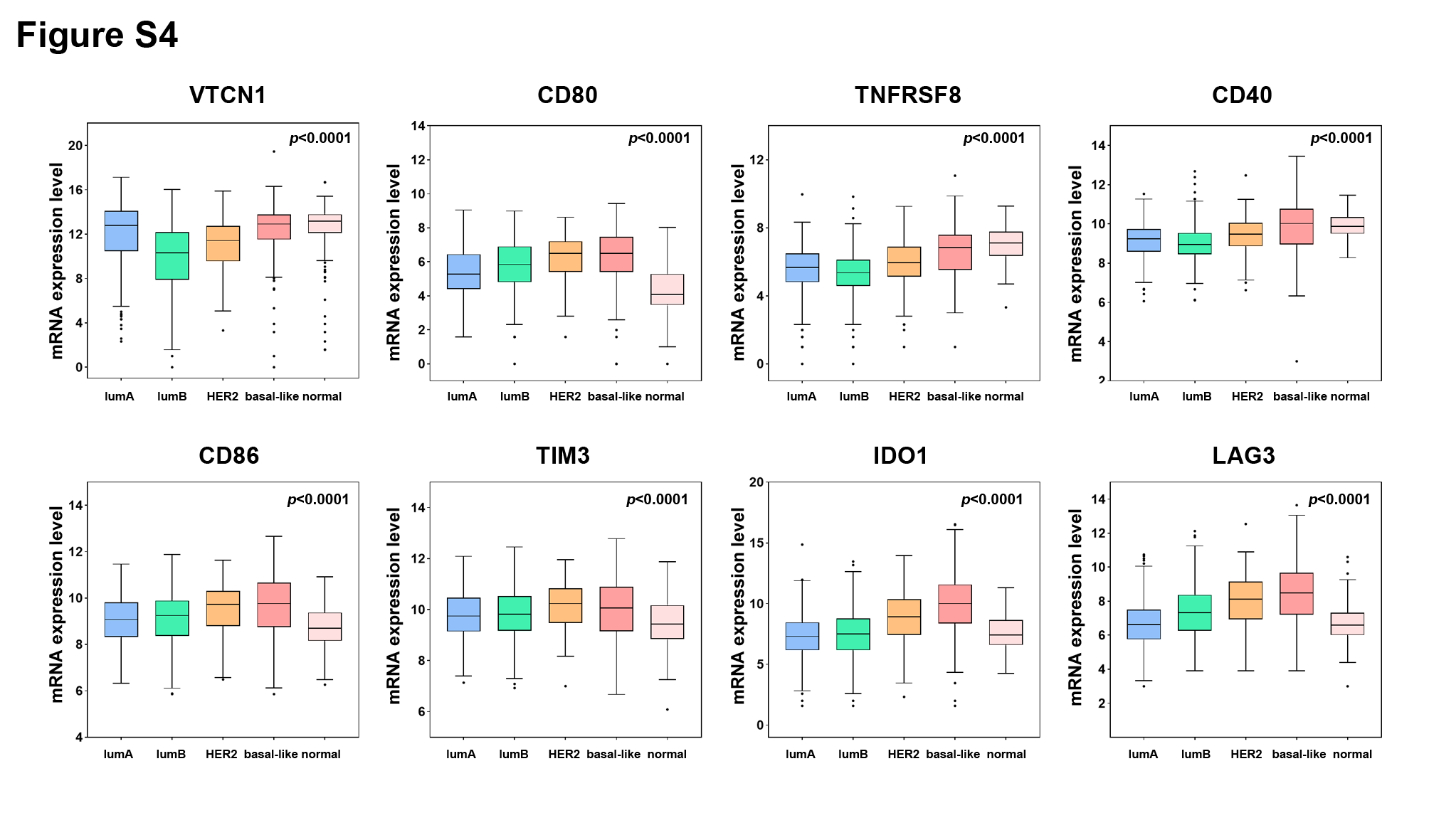

Supplement: Supplementary Figure 4 — Comparison of immune checkpoint gene mRNA expression levels among the breast cancer subtypes. [file Image_4.TIF]
